# Trapping MBD5 to understand 2q23.1 microdeletion syndrome

**DOI:** 10.15252/emmm.201404324

**Published:** 2014-07-07

**Authors:** Deborah Y Kwon, Zhaolan Zhou

**Affiliations:** Department of Genetics, University of Pennsylvania Perelman School of MedicinePhiladelphia, PA, USA

## Abstract

Despite genetic evidence implicating *MBD5* as the only overlapping gene between various 2q23.1 microdeletions, the function of MBD5 and its causality to 2q23.1 microdeletion syndrome, a disorder characterized by developmental delay and autistic features, has yet to be determined. In this issue of *EMBO Molecular Medicine*, Camarena *et al* generate an *Mbd5* gene-trap mouse model and show for the first time that mice with reduced MBD5 expression develop behavioral abnormalities with neuronal function deficits, mimicking symptoms in 2q23.1 microdeletion syndrome, thus supporting a causal role for *MBD5* haploinsufficiency in the disorder.

Chromosomal aberrations have been implicated in numerous developmental disorders. Among those is the 2q23.1 region, in which deletions and duplications lead to neurological disorders characterized by autism features and developmental delay (Mullegama *et al*, 2014). Patients with microdeletions in 2q23.1 typically present with intellectual disability, motor dysfunction, seizures, microcephaly, and craniofacial abnormalities (van Bon *et al*, 2010). The size of these deletions is highly variable and includes several previously identified disease-risk genes. Alignment of the deletion regions revealed a single overlapping locus spanning the methyl-CpG-binding domain 5 (*MBD5*) gene. This finding, accompanied by decreased *MBD5* mRNA expression in patient samples, suggested that *MBD5* haploinsufficiency is responsible for the observed disease manifestations (Talkowski *et al*, 2011; Williams *et al*, 2010). However, a causal role for *MBD5* in 2q23.1 microdeletion syndrome has yet to be established.

MBD5 is a member of the methyl-CpG-binding domain (MBD) family of proteins, which includes MBDs 1 to 6, MeCP2, SETDB1, SETDB2, BAZ2A, and BAZ2B (Klose & Bird, 2006). These proteins share an MBD domain, a conserved region of about 70 amino acids, and are believed to recognize DNA methylation and mediate chromatin modification and gene regulation, except MBD3, MBD5, and MBD6 that show weak binding to methylated DNA (Laget *et al*, 2010). MBD5, however, also contains a PWWP domain that recognizes histone methylation (Wu *et al*, 2011). Previous studies demonstrated that loss of the proteins that serve to read and interpret DNA methylation has deleterious effects and been linked to several neurodevelopmental disorders. Mutations in *MBD1*, *MBDs 3 to 6*, *SETDB1,* and *SETDB2* have been genetically implicated in autism (www.gene.sfari.org), and mutations in *MECP2* are responsible for Rett syndrome, a neurodevelopmental disorder that shares features with autism (Chahrour & Zoghbi, 2007). Notably, mutations in *MBD5* have been estimated to contribute to up to 1% of all autism spectrum disorder cases (Talkowski *et al*, 2011). However, the function of MBD5 and its mechanism of action remain virtually unknown.

In this issue, Camarena and colleagues set out to test the functional significance of MBD5 and its relationship to 2q23.1 microdeletion syndrome (Camarena *et al*, 2014). The expression pattern of murine *Mbd5* was found to be similar to that of human *MBD5,* and both are expressed in multiple isoforms in the brain. To examine the phenotypic consequences of *Mbd5* deficiency, the authors generated an *Mbd5* gene-trap mouse (*Mbd5*^*GT*^), in which a gene-trap cassette was inserted into intron 2 of *Mbd5* in order to disrupt protein production. This inclusion also allowed them to use X-gal as a proxy to examine MBD5 expression at a cellular level. Interestingly, they observed high X-gal expression in early embryonic stages that was primarily limited to neurons. Unexpectedly, the gene-trap cassette did not completely disrupt the production of full-length *Mbd5* mRNA, resulting in a hypomorphic model of MBD5. Animals homozygous for the gene-trap cassette with significantly reduced levels of *Mbd5* mRNA (*Mbd5*^*GT/GT*^) died perinatally, consistent with a previously published study showing pre-weaning lethality in *Mbd5*-null mice (Du *et al*, 2012). Consequently, heterozygous (*Mbd5*^*+/GT*^) mice were used to study the phenotypic effect of *Mbd5* haploinsufficiency.

their mutant mice developed a phenotype reminiscent of the impairments seen in 2q23.1 microdeletion syndrome patients

To determine whether MBD5 reduction in mice could recapitulate some of the clinical manifestations characteristic of 2q23.1 microdeletion syndrome, *Mbd5*^*+/GT*^ mice were subjected to a battery of physical and behavioral tests. Remarkably, their mutant mice developed a phenotype reminiscent of the impairments seen in 2q23.1 microdeletion syndrome patients. *Mbd5*^*+/GT*^ mice were found to be smaller than their wild-type littermates and many developed craniofacial abnormalities resulting from the irregular growth of the nasal bone. Behavioral analysis of the mutant mice revealed an array of discernible abnormalities, including impaired motor coordination, aberrant social behavior, excessive self-grooming, and reduced freezing during contextual and cued fear conditioning, indicative of impairment in emotional learning and memory. These features are thought to model some of the hallmarks of autism-like behavior and intellectual disability. However, the presence of seizures, another distinguishing symptom of 2q23.1 microdeletion syndrome, was not reported in these mice.

MBD5 may activate gene transcription to influence neuronal maturation and contribute to the behavioral phenotypes observed in *Mbd5*^*+/GT*^

To address how reduced expression of MBD5 might cause the observed neurological phenotypes, Camerena and colleagues cultured cortical neurons isolated from *Mbd5*^*+/GT*^ embryos and observed a distinct cellular phenotype. Neurite length and branching in *Mbd5*^*+/GT*^ neurons were reduced compared to neurons from wild-type mice, suggesting a role for MBD5 in proper neuronal development. To ascertain how MBD5 might be functioning, the authors took a reporter approach and performed a luciferase assay, finding that MBD5 could act as a transcriptional activator. Consistent with this result, immunocytochemistry experiments showed that MBD5 localizes to non-heterochromatin regions of the nucleus, in contrast to heterochromatin-associated MeCP2, an MBD family member known to mediate gene silencing. Although their approach is limited in reflecting the endogenous function of MBD5, these findings suggest that MBD5 may act differently from other MBDs in regulating downstream gene activity.

Taken together, the authors of this paper have developed a valuable genetic tool with the potential to dissect the pathogenic mechanisms of 2q23.1 microdeletion syndrome. Their mouse model is also useful in studying the function of MBD5, of which very little is known despite its clinical relevance. How MBD5 may activate gene transcription to influence neuronal maturation and contribute to the behavioral phenotypes observed in *Mbd5*^*+/GT*^ mice has yet to be determined, although their model is one significant step in advancing our knowledge of this devastating disorder. Interestingly, both duplications and deletions in *MBD5* have been linked to neurodevelopmental abnormalities (Mullegama *et al*, 2014). Likewise, deletions and duplications of *MECP2* give rise to the neurodevelopmental disorders, Rett Syndrome and *MECP2* Duplication Syndrome (Chahrour & Zoghbi, 2007), suggesting that proper dosage of MBD genes is critical for normal neurological development. This observed dosage effect would determine how future therapies are developed, as attaining a precise amount is of critical importance.

In addition to the brain, MBD5 is also expressed in peripheral tissues, particularly in the heart and kidney. The generation of conditional knockouts is needed to further delineate the function of MBD5 in these specific tissues and may circumvent the perinatal lethality seen in homozygous-null animals. Understanding the downstream effect of *MBD5* haploinsufficiency is another significant challenge. Given that MBD5 is highly expressed in neurons, the identification of its downstream gene targets is a considerably difficult task due to the complex cellular heterogeneity of the brain. Thus, future work is needed to establish how MBD5 functions in each neuronal type and to determine which are most vulnerable to its loss or overexpression. These studies may shed light not only on the cellular origins of 2q23.1 microdeletion syndrome but also other related neurodevelopmental disorders and may provide much needed avenues of therapeutic development.
